# Bone Defect Repair Using a Bone Substitute Supported by Mesenchymal Stem Cells Derived from the Umbilical Cord

**DOI:** 10.1155/2020/1321283

**Published:** 2020-04-05

**Authors:** Michal Kosinski, Anna Figiel-Dabrowska, Wioletta Lech, Lukasz Wieprzowski, Ryszard Strzalkowski, Damian Strzemecki, Lukasz Cheda, Jacek Lenart, Krystyna Domanska-Janik, Anna Sarnowska

**Affiliations:** ^1^Translational Platform for Regenerative Medicine, Mossakowski Medical Research Centre, Polish Academy of Sciences, Poland; ^2^Department of Stem Cell Bioengineering, Mossakowski Medical Research Centre, Polish Academy of Sciences, Poland; ^3^Paediatric Surgery Clinic, Institute of Mother and Child, Poland; ^4^Electron Microscopy Platform, Mossakowski Medical Research Centre, Polish Academy of Sciences, Poland; ^5^Department of Experimental Pharmacology, Mossakowski Medical Research Centre, Polish Academy of Sciences, Poland; ^6^Faculty of Chemistry, Biological and Chemical Research Centre, University of Warsaw, Poland; ^7^Department of Neurochemistry, Mossakowski Medical Research Centre, Polish Academy of Sciences, Poland

## Abstract

**Objective:**

Bone defects or atrophy may arise as a consequence of injury, inflammation of various etiologies, and neoplastic or traumatic processes or as a result of surgical procedures. Sometimes the regeneration process of bone loss is impaired, significantly slowed down, or does not occur, e.g., in congenital defects. For the bone defect reconstruction, a piece of the removed bone from ala of ilium or bone transplantation from a decedent is used. Replacement of the autologous or allogenic source of the bone-by-bone substitute could reduce the number of surgeries and time in the pharmacological coma during the reconstruction of the bone defect. Application of mesenchymal stem cells in the reconstruction surgery may have positive influence on tissue regeneration by secretion of angiogenic factors, recruitment of other MSCs, or differentiation into osteoblasts. *Materials and Methods*. Mesenchymal stem cells derived from the umbilical cord (Wharton's jelly (WJ-MSC)) were cultured in GMP-grade DMEM low glucose supplemented with heparin, 10% platelet lysate, glucose, and antibiotics. *In vitro* WJ-MSCs were seeded on the bone substitute Bio-Oss Collagen® and cultured in the StemPro® Osteogenesis Differentiation Kit. During the culture on the 1st, 7th, 14th, and 21st day (day in vitro (DIV)), we analyzed viability (confocal microscopy) and adhesion capability (electron microscopy) of WJ-MSC on Bio-Oss scaffolds, gene expression (qPCR), and secretion of proteins (Luminex). *In vivo* Bio-Oss® scaffolds with WJ-MSC were transplanted to trepanation holes in the cranium to obtain their overgrowth. The computed tomography was performed 7, 14, and 21 days after surgery to assess the regeneration.

**Results:**

The Bio-Oss® scaffold provides a favourable environment for WJ-MSC survival. WJ-MSCs in osteodifferentiation medium are able to attach and proliferate on Bio-Oss® scaffolds. Results obtained from qPCR and Luminex® indicate that WJ-MSCs possess the ability to differentiate into osteoblast-like cells and may induce osteoclastogenesis, angiogenesis, and mobilization of host MSCs. In animal studies, WJ-MSCs seeded on Bio-Oss® increased the scaffold integration with host bone and changed their morphology to osteoblast-like cells.

**Conclusions:**

The presented construct consisted of Bio-Oss®, the scaffold with high flexibility and plasticity, approved for clinical use with seeded immunologically privileged WJ-MSC which may be considered reconstructive therapy in bone defects.

## 1. Introduction

Bone defects resulting from a birth defect, injury, or ongoing disease processes often require reconstruction. So far as a standard procedure, own bone transplants were used. This means an additional procedure and sometimes health complications for the patient. According to scientific studies, such bone transplants undergo more often atrophy than tested biomaterial scaffolds. By introducing the bone scaffold into the human body, it is assumed that it will perform a specific function for a long time. Good anastomosis of the implant with the bone and its proper elasticity could create conditions that accompany the normal healing process of bone defect.

One of the biomaterials commonly used in stomatology is Bio-Oss® manufactured by Geistlich Pharma AG. This material is approved for clinical use in orthodontic surgeries. Bio-Oss® is composed of bovine bones deprived from lipids, blood components, and proteins; due to that after transplantation, graft rejection does not occur. Bio-Oss® has very similar structure to human cancellous bone, is flexible, and is elastic with high porosity which allows for cell adhesion and survival.

In recent years, biomedical field shows high interest in mesenchymal stem cells as a potential booster of endogenous regeneration of tissues. MSC expresses surface markers such as CD73, CD90, and CD105 and has potency to renewing and differentiating into preferred cell types such as bone and fat cells as well as chondrocytes. Every year, a number of clinical trials with MSC isolated from the bone marrow or adipose tissue increase. The role of those cells is not fully explained, but in the skeletal system, dermatology and ophthalmology are based on differentiation into targeted cell lines as well as on immunomodulatory and proangiogenic functions [1].

Mesenchymal stem cells were firstly isolated from the bone marrow; since then, those cells were characterized extensively and used frequently. Except the bone marrow, MSCs are isolated from the adipose tissue and umbilical cord. The number of isolated MSCs varies from 0.001 to 0.01% of total cells obtained from the bone marrow aspirate, approx. 2% in case of adipose tissue to approx. 25% in Wharton jelly of the umbilical cord [2]. Collection of the bone marrow as well as adipose tissue is associated with invasive procedures in contrast to the umbilical cord which is a waste during babies' delivery. Moreover, there are additional benefits from usage of fetal sources of MSC stem cells for regeneration purposes due to their expansive growth and higher spectrum of differentiation [3]. WJ-MSC is characterized by great plasticity and can be differentiated into bone and fat cells and chondrocytes and into sweat gland cells [4], Schwann cells [5, 6], and pancreas cells [7] or even neural-like cells [8]. Cells isolated from adult tissues due to longer exposure to environmental conditions may be characterized by reduced proliferation and regeneration potency and faster ageing what is connected to shorter telomeres. Compared to those cells, MSC from the umbilical cord has primary potency and unchanged properties due to its fetus origin [9].

The very important advantage of the WJ-MSCs is their low immunogenicity, which allows the use of those cells not only in autologous but also in allogeneic transplants with minimal risk of rejection. WJ-MSC is characterized by low protein expression of primary histocompatibility class I (MHC-I) and lack of MHC-II; thus, they are protected against lysis by NK cells [10]. Low immunogenicity is probably also associated with the lack of CD40, CD80, and CD86 as immunologic response costimulants on the surface of cells and simultaneous expression of its inhibitors—indoleamine 2,3-dioxygenase (IDO) and prostaglandin E2. In addition, WJ-MSC expresses human leukocyte antigens HLA-G5 (human leucocyte antigen G5) and HLA-G6, which are involved in the process of fetal tolerance in the mother's uterus by inhibiting lymphocyte proliferation. WJ-MSC does not form teratomas after transplantation into mice with weakened immune systems, and so far, no such cases have been reported in patients whom WJ-MSCs were transplanted [11, 12].

## 2. Materials and Methods

**Scheme 1:**
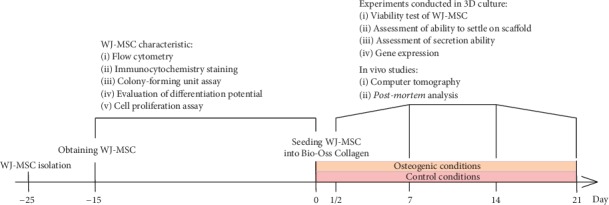
Schematic illustration of the conducted experiments.

Our experiments were conducted according to Scheme 1.

### 2.1. Isolation and Culture of WJ-MSC

The umbilical cords (UC) were taken from the University Medical Center for Women and the Newborn of the Medical University of Warsaw with consents of mothers and Local Ethical Comity. The UCs were transported in PBS with 100 j/ml penicillin and streptomycin and 0.25 *μ*g/ml amphotericin B. Isolation procedures were performed in less than 24 h after birth. The UCs were sliced into 1-2 mm pieces. 2 mm biopsy pieces were taken from perivascular zone of Wharton's jelly and placed on 6-well cell culture plates. Tissues were cultured in 90% DMEM (Macopharma), 10% platelet lysate (Macopharma), 2 j/ml heparin, 1% (*v*/*v*) antibiotics (Gibco), 1% (*v*/*v*), and 100 mg/ml glucose in 5% O_2_. Medium was changed every 2-3 days. Cells migrated out from tissue pieces were harvested with Accutase (BD).

### 2.2. Flow Cytometry Analysis

To determine MSC markers, cells were harvested from cell culture flask with Accutase. Cells were stained by mouse anti-human antibodies conjugated with fluorochromes: fluorescein isothiocyanate (FITC), phycoerythrin (PE), peridinin-chlorophyll-cyanine protein complex (PerCP-Cy™), or allophycocyanin (APC): CD90-FITC, CD44-PE, CD105-PerCP-Cy™, CD73-APC (BD Stemflow™ hMSC Analysis Kit). Cells were analyzed by a FACSCalibur II (Becton Dickinson).

### 2.3. Immunocytochemical Analysis

Cells were seeded onto coverslips with polylysine in 24-well cell culture plate. Cells were fixed with 4% paraformaldehyde for 10 min, permeabilized with Triton X-100 (Sigma-Aldrich) for 15 min (not apply when stained for CD73 and CD90), and blocked with 10% goat serum (10% donkey serum when stained for osteopontin) with 1% bovine serum albumin (Sigma-Aldrich) for 1 h. Cells were incubated with mouse anti-human primary antibodies CD73, CD90, osteocalcein, goat anti-human osteopontin, and rabbit anti-mouse primary antibodies Ki67 overnight in 4°C (Table 1). Antibodies were washed few times with PBS and secondary antibodies goat anti-mouse/rabbit or for osteopontin donkey anti-goat antibodies were added for 1 h in RT. Cell nuclei were counterstained with Hoechst 33342. Immunocytochemical staining for osteocalcein was performed on freshly isolated cells and 21 days after osteodifferentiation and examined under a fluorescent microscope Zeiss AxioVert.A1. Immunocytochemical staining for osteopontin was performed on cells cultured 21 days on Bio-Oss® Collagen scaffold in osteodifferentiation medium and examined under a confocal microscope Zeiss LSM780.

### 2.4. Colony-Forming Unit Assay

The umbilical cord was cut into half. One part was frozen in 80% PBS, 10% 2 M sucrose, and 10% DMSO. The second one was used for fresh isolation. WJ-MSCs on the 4^th^ passage were seeded 10 cells/well on 6-well cell culture plate in duplicates. After 10-12 days, cells were fixed with 4% paraformaldehyde for 15 min and stained with 0.5% solution of toluidine blue for 20 min in RT. Cells were examined under confocal microscope Zeiss LSM780. The number of CFU-F was counted in ZEN 2.3 lite software. After 1-month storage of frozen tissue, the UC was thawed and all steps were conducted as for fresh UC. Experiment was performed in 3 repetitions.

### 2.5. Osteogenic, Chondrogenic, and Adipogenic Differentiation of WJ-MSC

Cells were seeded in a 24-well cell culture plates at a density of 5 × 10^3^ cells/cm^2^ for osteogenesis and 1 × 10^4^ cells/cm^2^ for adipogenesis. After reaching 50-60% confluency of WJ-MSC for osteogenesis and 70% confluency for adipogenesis, standard growth medium was replaced by StemPro® Osteogenesis/Adipogenesis Differentiation Kit. For chondrogenesis, 1 *μ*l droplets of 1.6 × 10^7^ cells/ml solution were seeded in the centre of the wells, and after 10 min in high-humidity conditions, the StemPro® Chondrogenesis Differentiation Kit was gently added. After 21 days of culture in osteodifferentiation medium, 14 days in adipodifferentiation medium, and 14 days of chondrodifferentiation medium, cells were fixed in 4% paraformaldehyde and stained with Alizarin Red S, Oil Red O, and 1% Alcian Blue solution, respectively. When cells were cultured in differentiation mediums, cell culture plates were transferred into 21% O_2_ as recommended by the manufacturer.

### 2.6. Cell Proliferation Assay

WJ-MSCs were seeded 3000 cells/cm^2^ in 96-well cell culture plates. Cells were cultured in standard culture medium and in StemPro® Osteogenesis Differentiation Kit for 8 days, 6 wells for each condition, one plate for each day. After 19.5 h ± 0.5 h, mediums were changed onto DMEM w/o phenol red with platelet lysate, heparin, glucose, and antibiotics. 10 *μ*l of MTT salt was added into the wells and incubated for 4.5 h. 25 *μ*l of solution was left in the wells and 50 *μ*l of DMSO was added. Absorbance was measured in FLUOstar Omeg (BMG Labtech). Procedure was repeated every day for 8 days. Population doubling time was obtained for each condition according to the following formula: (*t* − *t*_0_) × log2/(log*N* − log*N*_0_) where *t* is the day of experiment, *t*_0_ is the initial day of experiment, *N* is a number of cells in a particular day, and *N*_0_ is an initial number of cells in *t*_0_.

### 2.7. Seeding Cells into the Scaffold

Bio-Oss® Collagen was purchased from Geistlich Pharma SA. 100 mg cube was cut into 16 smaller pieces by scalpel. 30 *μ*l of 8,000,000 cells/ml cell solution was injected into the scaffolds. Scaffolds with cells were transferred into 24-well cell culture plates, with a maximum of 3 cubes into 1 well. Bio-Oss® Collagen with WJ-MSC was cultured in standard culture medium and in StemPro® Osteogenesis Differentiation Kit for 21 days. After 24 h, scaffolds were transferred into new wells.

### 2.8. Cell Viability Test on Bio-Oss® Collagen

Cells were seeded into the scaffolds as mentioned before. Cell viability test was performed on culture in standard culture medium and StemPro® Osteogenesis Differentiation Kit in days 1, 7, 14, and 21. In each time point, scaffolds with cells were stained by ethidium homodimer-1 and calcein AM in DMEM for 20 min in RT. After washing with PBS, scaffolds were examined under confocal microscope Zeiss LSM780 in *Z*-plane from which 2D pictures were generated.

### 2.9. Assessment of the Settlement of WJ-MSC on Bio-Oss® Collagen

Cells were seeded into the scaffolds according to Seeding Cells into the Scaffold. Assessment was performed in days 1, 7, 14, and 21. For each time point and condition, the scaffold without cells was prepared as a negative control. In each time point, scaffolds were transferred into fixation solution for electron microscopy. Scaffolds were examined under a scanning electron microscope JSM-6390LV (JEOL).

### 2.10. WJ-MSC Protein Secretion in 3D Culture

Cells were seeded into the scaffolds according to Seeding Cells into the Scaffold, There are 3 scaffolds for each time point in each condition. In days 2, 7, 14, and 21, medium was collected from all wells and coupled into one falcon tube for each condition, frozen and stored at -80°C. 24 h before collection, mediums were changed into fresh ones. Standard culture medium and StemPro® Osteogenesis Differentiation Kit were collected as a negative control. Experiment was conducted in 3 repetitions. All mediums were thawed and centrifuged in 500 x g for 5 min. 3 ml of each supernatant was transferred into Spin-X 6 UF concentrators (SIGMA) with cut-off 5 kDa and centrifuged 1800 x g for 15 min. BMP-2, FGF-23, IL-6, osteoprotegerin, RANKL, Dkk-1, IL-1*α*, osteopontin, TNF-*α*, VEGF-D, and TGF-*β*1 were analyzed in Luminex® assay. All steps were conducted following the manufacturer's instructions. Luminex® plates were read in Luminex Bio-Plex® 200 System.

### 2.11. RNA Isolation and qPCR Analysis

Cells were seeded into the scaffolds as mentioned before, 3 scaffolds for each time point in each condition. In days 2, 7, 14, and 21, scaffolds were collected and suspended in 400 *μ*l of phenosol, frozen and stored at -80°C. Experiment was conducted in 3 repetitions. Total RNA was isolated from scaffolds with Total RNA Mini Plus (A&A Biotechnology), cleaned, and concentrated with Clean-Up RNA Concentrator (A&A Biotechnology) following the manufacturer's instructions. 19 ng of RNA from each probe was reverse-transcribed using RNA-to-cDNA kit (Thermo Fisher) following the manufacturer's instructions. cDNA was preamplified with SsoAdvanced PreAmp Supermix following the manufacturer's instructions. 25 *μ*l of cDNA was added into 225 *μ*l of water for each probe. 5 *μ*l of cDNA solution, primers, and a mix from 3color RT HS-PCR Mix Sybr® were used for each qPCR reaction. PCR was performed with specific primers for human *GAPDH*, *B2M*, *RPLI3A*, *HPRT1*, *TBP*, *PPIA*, *BGLAP*, *ALPL*, *RUNX2*, *COL1A1*, *VDR*, and *SNAI1.* qPCR conditions are 300 s in 95°C, 40 cycles of 15 s in 95°C, and 60 s in 60°C. The reference gene was determined by geNorm and NormFinder. The reference gene was used: TBP. GeneEx 6.1 software (MultiD Analyses AB, Göteborg, Sweden) was used to analyze the data by the Pffafl method [13] (the quantification cycle (Cq) values and the baseline settings automatically calculated by the qPCR instrument software) from LightCycler® 96 Software (Roche Diagnostics GmbH, Mannheim, Germany) (Table 2).

### 2.12. In Vivo Transplantation

Four Wistar rats were used in the following experiments. Under anesthesia, 4 trepanation holes (~0.2 cm width) were made in the scalp of each rat. One was left empty, one was filled with Bio-Oss® Collagen, one was filled with Bio-Oss® Collagen with injected cells, one was filled with Bio-Oss® Collagen with injected cells, and additional injection of cell solution was applied on the place of transplantation. Rats were kept for 21 days after surgery.

### 2.13. Computed Tomography

After 7, 14, and 21 days, rats' scalps were examined in computed tomography under the anesthesia. Computed tomography was made with Albira PET/SPECT/CT Preclinical Imaging System (Bruker). In time of each imaging, rats get anesthetized with isoflurane (Baxter Polska Sp. z o.o.) in oxygen applied through a nose cone and respiration was monitored. CT scan parameters were set as follows: tube voltage was 45 kVp, tube current was 400 *μ*A, and number of projections was 1000. Minimal resolution of CT was 90 *μ*m. 3D reconstruction of scalps was made, and comparison of bone regeneration between time points was done in PMOD software, version 3.307, module View Tool [PBAS] (PMOD Technologies LLC). All procedures made on animals were approved by the First Local Ethical Committee of the Warsaw University Biology Department (Permission No. 560/2018).

### 2.14. Postmortem Analysis

After rats were sacrificed, cranial vaults were isolated and submerged into 2 j/ml of collagenase for 2 h in 37°C with shaking. Soft tissues were removed from the scalps and pictures were made with binocular with camera.

### 2.15. Statistics

Statistical analysis of the raw data was conducted using GraphPad Prism 5 software. The Kolmogorov-Smirnov test was used as a normality test. The Student *t*-test for a pair of group or one-way ANOVA followed by Tukey's multiple comparison test for comparison inside the group was used: ^∗^*p* < 0.05, ^∗∗^*p* < 0.01, ^∗∗∗^*p* < 0.001, and ^∗∗∗∗^*p* < 0.0001. Results represent three independent experiments, each in at least four replicates. Results presented at the graphs were shown as mean with standard.

## 3. Results

### 3.1. Isolation Efficiency and WJ-MSC Characteristic

**Scheme 2:**
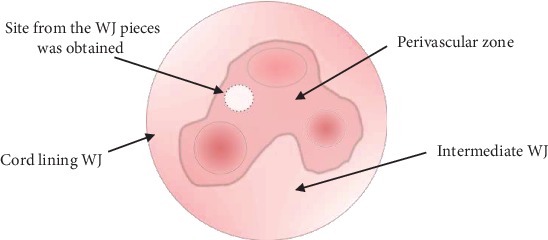
Cross section of the umbilical cord with a marked site from the Wharton's jelly (WJ) pieces was obtained.

WJ-MSCs were isolated from fresh and frozen UC tissue from Wharton's jelly obtained from perivascular zone (Scheme 2).

**Figure 1:**
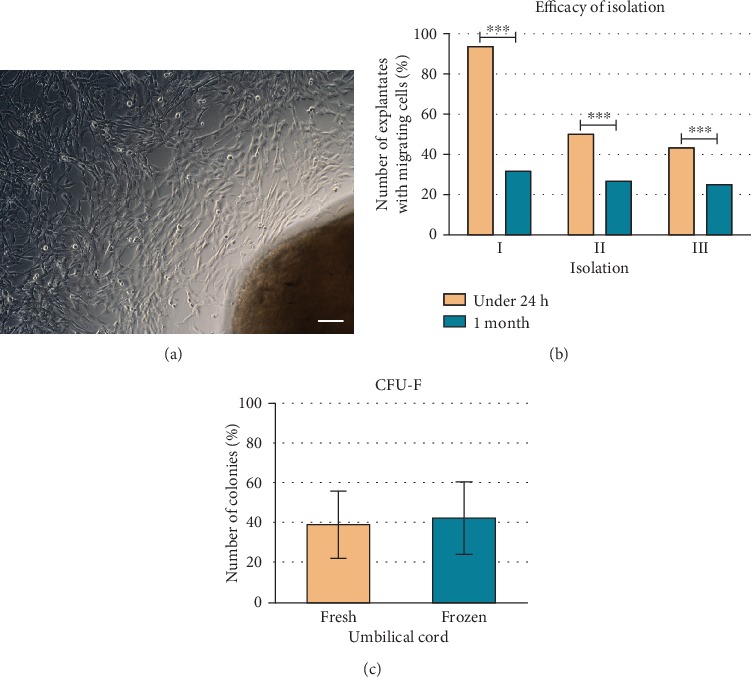
Picture from light microscopy of WJ-MSC migrated from Wharton's jelly explantate after 10 days in culture (a). Size of scale bar: 100 *μ*m. Number of explantates with migrating cells after isolation from the fresh and frozen umbilical cord in three repetitions (b). Number of colonies formed from single cells when 10 cells were seeded on a 6-well dish from cells derived from Wharton's jelly from the fresh and frozen umbilical cord (c).

Cells migrated out from tissue pieces showed typical fibroblast-like morphology (Figure 1(a)). Difference in efficiency of isolation between fresh and frozen UC was observed but did not affect ability to isolate WJ-MSC (Figure 1(b)). WJ-MSCs were able to form CFU-F from a single cell. In average, 40% of cells were capable of clonogenicity from fresh and frozen isolation (Figure 1(c)).

**Figure 2:**
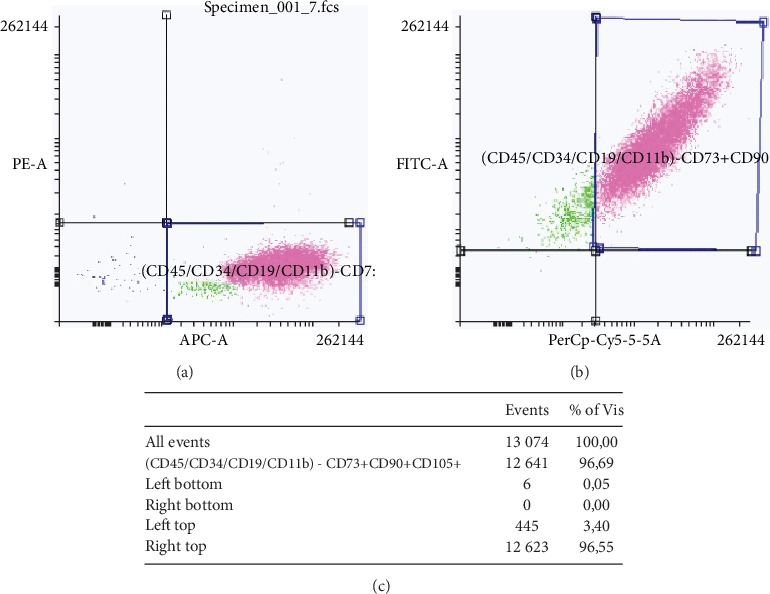
Flow cytometry analysis. Representative gating for CD45/CD34/CD19/CD11b-negative and CD73-positive markers (a) and for CD90- and CD105-positive markers (b). Table with results obtained from flow cytometry (c).

Flow cytometry analysis showed around 97% of isolated cells with expression of CD73, CD90, and CD105 with lack of CD45, CD34, CD19, and CD11b markers (Figures 2(a)–2(c)).

**Figure 3:**
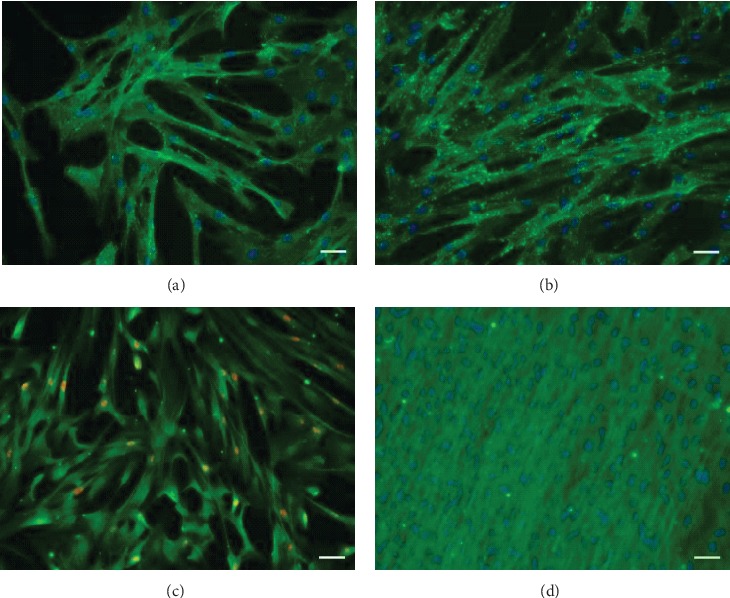
Immunocytochemistry staining of WJ-MSC. CD73 (green; a), CD90 (green; b), osteocalcein (green; c, d), and Ki67 staining (red; c). (a, b, d) Nuclei were counterstained with Hoechst 33342. Size of scale bar: 50 *μ*m.

Immunocytochemical staining showed CD73 and CD90 expressions in WJ-MSC. In few cells, osteocalcein was observed in undifferentiated cells but not the osteopontin. After osteodifferentiation, WJ-MSC expressed in 89.6 ± 7% osteocalcein. In undifferentiated cells, great number of Ki67 expression was observed (Figure 3).

**Figure 4:**
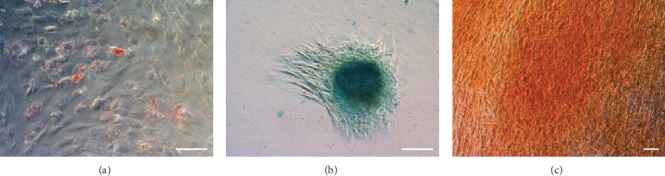
Evaluation of differentiation potential of WJ-MSC. WJ-MSC differentiated into adipocytes (a), chondrocytes (b), and osteocytes (c). Cell cultures were stained with Oil Red O, Alcian Blue, and Alizarin Red S, respectively. Size of scale bar: 100 *μ*m.

WJ-MSCs differentiated into adipocytes, chondrocytes, and osteocytes which was confirmed by positive staining for Oil Red O, Alcian Blue, and Alizarin Red, respectively (Figure 4).

**Figure 5:**
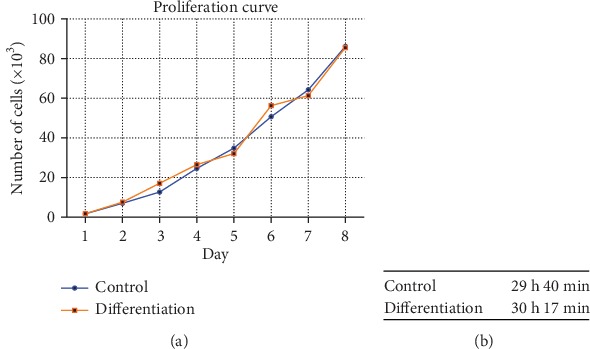
Cell proliferation analysis. Graph represents the proliferation curve for cells cultured in control medium and cultured in osteodifferentiation medium during 8 days of culture (a). Mean of the doubling time is written in the table for control and for differentiation conditions (b). Doubling time was calculated based on the following equation: (*t* − *t*_0_) × log2/(log*N* − log*N*_0_).

There was no noticeable difference in doubling time of WJ-MSC from day 1 to day 8 for cells in standard culture medium and in osteodifferentiation medium which was approximately 30 h (Figure 5).

### 3.2. Cell Viability Test and Assessment of the Settlement of WJ-MSC on Bio-Oss® Collagen

**Figure 6:**
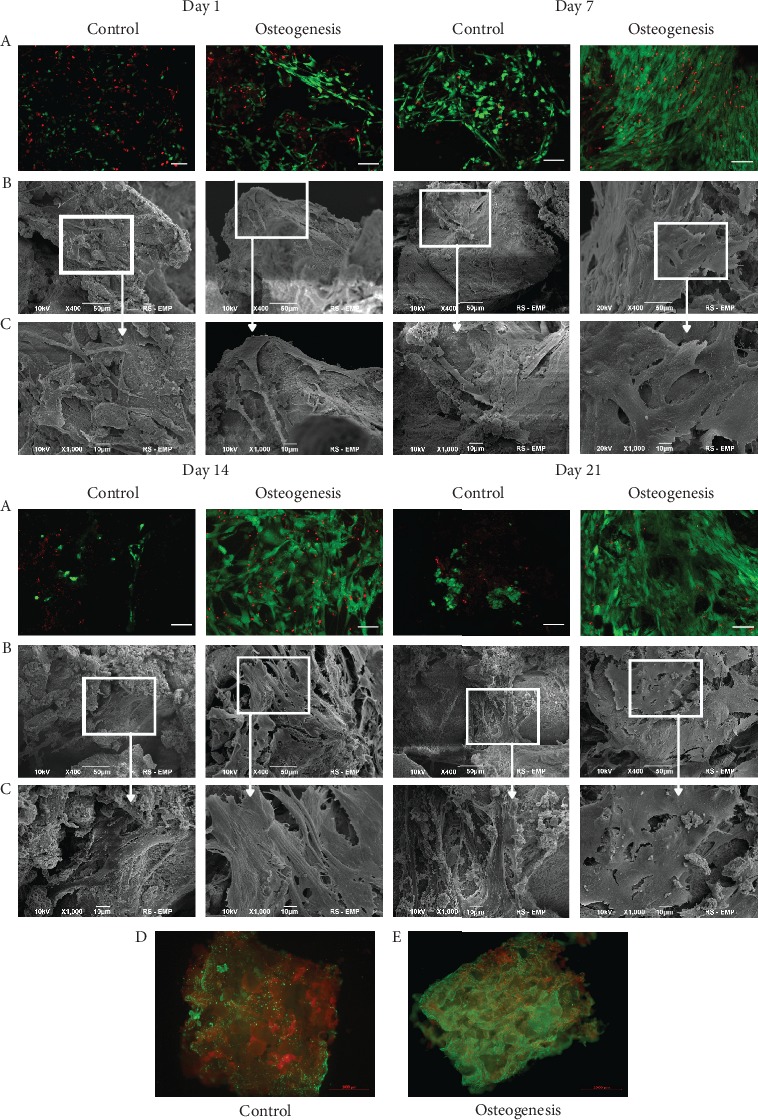
WJ-MSC on the Bio-Oss® Collagen scaffold cultured in control medium and osteodifferentiation medium. Pictures from a confocal microscope (a)—cells were stained with calcein AM (alive cells—green staining) and ethidium homodimer (dead cells—red staining). Pictures from an electron microscope (b, c). Scale bar in (a) row: 100 *μ*m. Image of the entire scaffold cultured with WJ-MSC in control (d) and osteodifferentiation (e) medium (green—calcein AM; red—ethidium homodimer). Scale bar: 1000 *μ*m

On the first day in both conditions, viability of the cells on Bio-Oss® Collagen was comparable, but in osteodifferentiation, medium cells had fibroblast-like morphology. On the 7^th^ day, viability of cells was similar in both conditions. In osteodifferentiation, medium cells become shorter and wider and approximately whole surface of the scaffold was covered by a layer of cells compared to few cells attached to scaffold in control conditions. Boundaries between cells in osteodifferentiation conditions are blurred making hard to distinguish separate cells. Until the end of experiments, similar observations were noticed with even a greater reduction of the number of cells in control condition and more osteoblast-like morphologies in osteodifferentiation condition (Figure 6).

**Figure 7:**
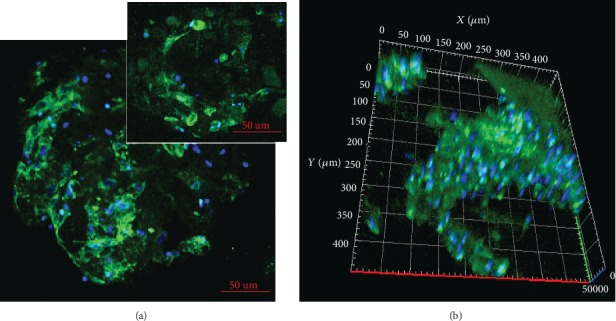
The immunocytochemical analysis of osteopontin (green) expression in WJ-MSC cultured on the Bio-Oss® Collagen scaffold after osteodifferentiation. (a) Confocal imaging shows osteopontin expression in single cells; specific staining was localized to the cytoplasm. (b) 3D cross-sectional image illustrates the distribution of WJ-MSC expressing osteopontin inside the skeleton. The nuclei were stained with Hoechst (blue). Scale bar: 50 *μ*m.

Immunocytochemical staining of WJ-MSC cultured on Bio-Oss® Collagen showed osteopontin expression in those cells (Figure 7). WJ-MSC expressed osteopontin in 47.3 ± 13%.

### 3.3. Protein Secretion in 3D Culture

**Scheme 3:**
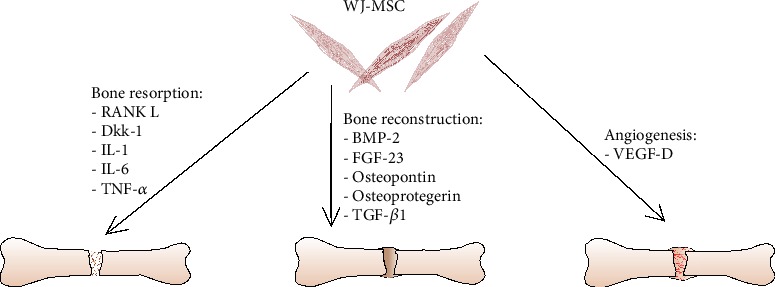
Schematic illustration of cytokines involved in the process of bone regeneration secreted by WJ-MSC.

WJ-MSC secretes cytokines which are crucial during bone regeneration process (Scheme 3). From days 2 to 21 in osteodifferentiation medium, increase in protein concentration of BMP2 secreted by seeded cells can be observed. In control medium, this tendency does not occur. Similar changes in protein concentration were observed for FGF-23 which level increased until day 7^th^ when reached plateau only in osteodifferentiation medium.

For VEGF in control medium, protein amount decreases from the 2^nd^ day to the 14^th^ day. Reverse results can be observed in differentiation medium where from the 2^nd^ day to the 7^th^ day, concentration increases and holds that level until the end of the experiment.

In control conditions, osteopontin concentration increased in the 2^nd^ day followed by decrease of protein level through next time points, whereas in osteodifferentiation medium, constant increase in protein amount was noticed from the beginning until the end of the experiment.

Osteoprotegerin concentration slightly increased in control condition through a time, but in differentiation medium, great increase can be observed from the 7^th^ day and its stable secretion until the 21^st^ day.

In control conditions, a slight decrease of RANKL level can be observed from days 2 to 21, whereas in osteodifferentiation medium, a 2.5-fold increase is observed in day 7 and this level is stable until the end of experiment.

In both conditions, tendency to increased secretion of TGF-*β*1 from days 2 to 21 can be observed.

In both conditions, in day 2, great amounts of IL-6 were secreted and in the next day level, this protein drastically dropped.

Through 21 days of experiments in control conditions, secretion of Dkk-1 is almost not observed compared to a great amount and its tendency to increasement of protein level in osteodifferentiation medium until the last day.

**Figure 8:**
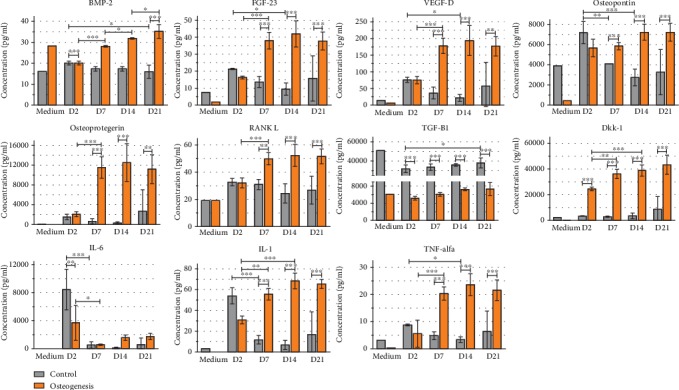
Luminex analysis of protein secretion produced by WJ-MSC cultured in control medium and osteodifferentiation medium in days 2, 7, 14, and 21 cultured on 3D scaffolds. “Medium” bars represent the basic level of proteins in the medium.

Concentration of IL-1 and TNF-*α* in control conditions decreased from day 2. In osteodifferentiation conditions, reverse tendency can be observed and until day 7 increasement of protein level can be observed and then secretion of those protein stabilizes on certain level until the end of experiment (Figure 8).

### 3.4. Gene Expression (qPCR)

**Figure 9:**
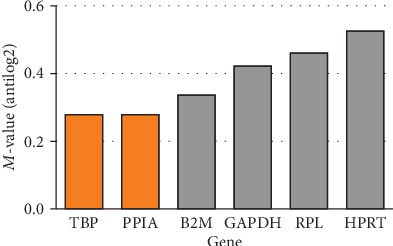
Analysis of reference genes for WJ-MSC by geNorm software.

After analysis of 6 reference genes, the two most stable genes were chosen by geNorm software—TATA binding protein (*TBP*) and peptidylprolyl isomerase A (*PPIA*) and one gene chosen by NormFinder software—*TBP* (Figure 9). For the next analysis, *TBP* gene was used as a reference gene.

**Figure 10:**
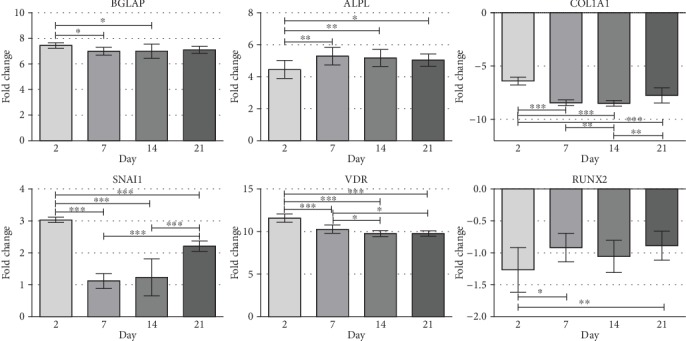
Canonical gene expression of BGLAP, ALPL, RUNX2, VDR, SNAI1, and COL1A1 during osteoblast differentiation of WJ-MSC cultured on scaffolds in osteodifferentiation medium obtained by the 2^−ΔΔCt^ method. The *y*-axis of each plot represents gene expression fold change, relative to day 0 (nondifferentiated cells). The *x*-axis represents the time point post-OB induction (day 2, day 7, day 14, and day 21). Error bars represent the standard error of the mean, and measurements are based on 3 replicates.

After procedures of RNA isolation from WJ-MSC cultured on Bio-Oss® Collagen in standard culture medium, there was shortage of material needed for qPCR. Due to that, comparison of gene expression in material from WJ-MSC cultured in 2D in standard culture medium was used. A 7-fold increase of *BGLAP* and a 5-fold increase of *ALPL* expression were observed from day 2 to day 21. A 6-fold decrease of *COL1A1* expression was observed in day 2 and 8-fold from days 7 to 21. On the day 2 of experiment, a 3-fold increase in expression was observed for *SNAI1*; on days 7 and 14, expression dropped to a 1-fold increase compared to control; on day 21, expression increased again to 2-fold. *VDR* expression elevated 10-folds from day 2 to day 21. Expression of *RUNX2* slightly decreased, approximately 1-fold during 21 days of experiment (Figure 10).

**Figure 11:**
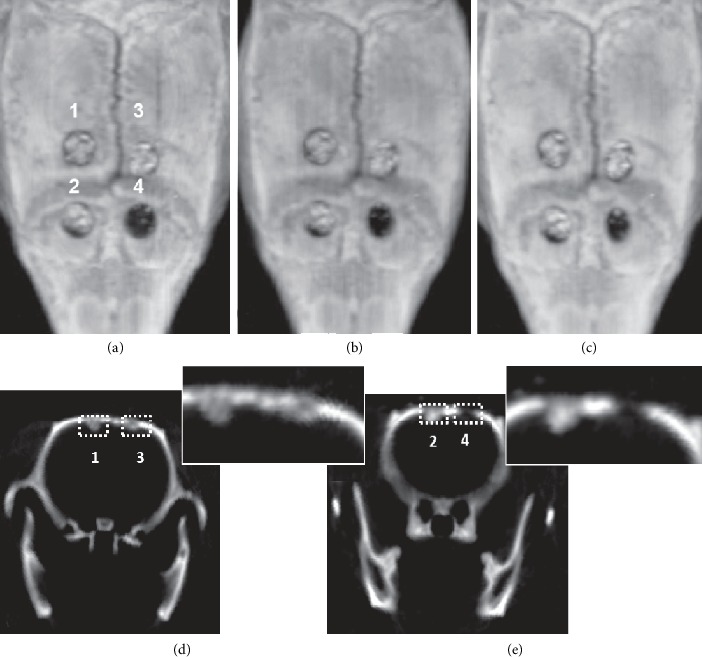
Computed tomography images of the rat scalp. (a–c) Transversal sections: 7 (a), 14 (b), and 21 (c) days after scaffold transplantation. 1—Bio-Oss® with injected WJ-MSC into and onto the scaffold; 2—Bio-Oss® with injected WJ-MSC; 3—Bio-Oss; 4—empty hole after trepanation (*n* = 4). (d, e) Cross-coronal sections.

Computed tomography scans on days 7, 14, and 21 show a lack of regeneration in place of empty hole after trepanation (Figure 11). In case of trepanations, scans on holes filled with scaffolds show radiological regeneration. Up to 21 days, there is no difference between variants of injected cells.

**Figure 12:**
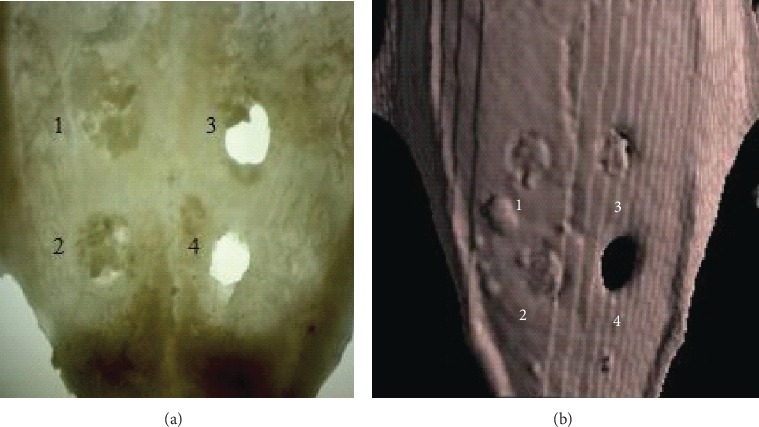
Cranial vaults of the rat skull after removal of soft tissues. 1—Bio-Oss® with injected WJ-MSC into and onto the scaffold; 2—Bio-Oss® with injected WJ-MSC; 3—Bio-Oss® alone; 4—empty hole after trepanation. (a) Light binocular. (b) Computed tomography.

After scalp incubation in collagenase solution and removal of soft tissues with gauze, scaffold without cells fell out from the hole. Scaffolds in which cells were injected show connection with bone tissue of the scalp (Figure 12).

## 4. Discussion

Wharton jelly (umbilical cord stroma) due to its fully noninvasive collection and high availability might be the most convenient source of MSC (WJ-MSC) for future clinical therapy. The advantage of cells isolated from the after-birthing is their early developmental stage, which makes them less differentiated, with higher proliferative potential than somatic MSC and with higher clonogenic abilities and a slower aging rate. Moreover, WJ-MSC shows negligible immunogenicity. This is due to the very low expression of MHC class I antigens (HLA-ABC) and the lack of MHC class II antigens (HLA-DR) and costimulatory antigens such as CD80 and CD86 involved in the activation of both T lymphocyte responses and B lymphocytes. Considering both the availability and the ability to administer cells in an allogeneic system, it seems that WJ-MSC might be the better source for clinical use than bone marrow-derived MSC. In order to obtain the largest possible amount of WJ-MSC with high proliferative and clonogenic potential and good survival, we have developed a protocol for isolation from specific regions of umbilical cord. Similarly to Subramanian group [14], we isolate cells with mechanical technique from the perivascular region. The best results were obtained using biopunch with 2 mm diameter.

Technique used in our laboratory for WJ-MSC isolation gives high yield of cells with their good quality. CFU-F of isolated cell population reach up to 40% which is a positive result compared to 10-30% MSC population reported by other scientists [2, 9, 15]. Wharton's jelly isolated with our method can be frozen without reduction of the MSC population after isolation what contradicts Chatzistamatiou who states that efficient isolation of MSC from umbilical cord is possible only from fresh tissue [16].

To date, MSC isolated from the bone marrow have been the most commonly used in orthopedic surgery. In our previous papers [8], we compared BM-MSC and WJ-MSC underlying some properties of WJ-MSC unique only for this fraction. WJ-MSC preferentially differ into mesodermal cell linages as Chen and Avercenc-Léger show in theirs works ([17, 18]), but its differentiation potency is not limited only to one germ layer. WJ-MSC can also differentiate toward cell linages from ectoderm and endoderm germ layer which was proven by Maher Atari and our team ([19–21]). Thus, plasticity of those cells shows their high therapeutic potential.

For bone reconstruction, transplanted cells must possess the ability to differentiate toward osteoblasts (OB). From the literature, there is known that these cells are recruited during remodeling from host stem/stromal mesenchymal cells. In some clinical situations, the number of host precursors is insufficient that is why human MSCs are now being introduced into the clinic.

MSCs play multidirectional function. In addition to osteoblast differentiation, MSC supports osteoclast development. Osteoclast development requires cell-to-cell contact between osteoclast progenitors which come from hematopoietic cells. Osteopontin seems to be the factor that plays a key role in this process. Osteopontin is expressed during the early stage of the differentiation of osteoblast and osteoclast progenitors and its cell adhesion properties are important for osteoclastogenesis. It is responsible for attachment of cells to matrix substances or other cells facilitating the interactions. Yamate et al. [22] described antiosteopontin antibody or RGD-containing peptide inhibits that process, whereas freshly isolated WJ-MSCs secrete osteopontin but the secretion significantly declines during cell culture; in differentiating conditions, its level is constantly increasing.

Moreover, osteopontin is thought to potentiate the secretive potential of MSC that in turn promote proliferation and differentiation of the hematopoietic precursors.

WJ-MSC isolated and cultured in described conditions can similar to BM-MSC contribute to bone regeneration directly by differentiation into osteoblast-like cells, and indirectly by stimulation of maturation of osteoblasts and osteoclasts as well as the activation of angiogenesis by secretion of VEGF-D.

Bone reconstruction takes place in several stages with different crucial factors (Scheme 3). The first stage concerns a resorption of the bone. During this stage, secretion of Dkk-1, IL-6, IL-1, and TNF-*α* increases what is a booster for osteoclastogenesis [23, 24]. In the next stage of regeneration, formation of the new bone takes place. BMP-2 accumulated in ECM [25] counteract with osteoblasts receptors which induce bone formation. During our experiments, secretion of BMP-2 increased. Similar tendency was observed for FGF-23, a protein which maintains homeostasis of ions and mineralization of the bone [26]. During 3D culture, cells were adherent to the scaffold, alive, and capable of migration. Those features were correlated with the increase of osteopontin secretion which also contributes to bone remodelling, stress response, and repair ([27]). Resorption and formation of the new bone have to be balanced. It is maintaining by osteoprotegerin (OPG) and RANKL. OPG is a competitive inhibitor of RANKL which is responsible for osteoclast maturation and bone resorption, appropriate ratio between OPG and RANKL which is necessary for maintaining proper bone volume [28, 29]. Both protein levels increased during experiments. For correct bone regeneration, a simultaneous angiogenesis is required. The supply of nutrients to cells that settle a damaged tissue fragment is critical to the bone regeneration process [30]. VEGF-D, which stimulates the proliferation and migration of endothelial cells, belongs to important proangiogenic factors. Despite the observations described by the Amable group, which found that WJ-MSCs do not secrete VEGF [31], during our experiments, we observed a significant increase in VEGF-D level in the differentiating medium from days 2 to 7 and maintaining this level until day 21. In line with my observations, the proangiogenic properties of WJ-MSC have also been described by Obtulowicz et al. and Widowati et al. [21, 32]. It can therefore be assumed that WJ-MSCs by stimulating VEGF-D production promote formation of new blood vessels and thus support and accelerate the regeneration of tissue (Scheme 3).

In the case of extensive defects in bone tissue, the use of cell therapy alone does not bring the expected results due to the lack of scaffolding needed to cell growth and tissue regeneration could occur. It is necessary to apply a combination therapy using bone graft material along with cells that will accelerate the regeneration of the structure and support the linking of the introduced material with the patient's bone through the above secretory, inductive, and differentiation properties of mesenchymal stem cells [33]. Numerous works have shown that the appropriately selected material from which the 3D skeleton is created may, due to its biophysical properties, e.g., elasticity, shape, or porosity, influence the differentiation of mesenchymal cells [34–36]. Unfortunately, sometimes the biochemical composition of such materials despite meeting the topographic conditions may prevent proper cell survival, adhesion, and differentiation. Some of the materials tested cannot be used for clinic; the others are not biodegradable. That is why research on selection of the optimal skeletons for the regeneration is still ongoing.

In our *in vitro* studies, we have shown that WJ-MSCs are able to colonize the Bio-Oss® Collagen scaffold, and the resulting construct is characterized by high cell survival and allows the formation of complex osteoblastic structures with a characteristic disappearance of the boundaries between individual cells. Moreover, MSC seeded into the scaffold and cultured in osteo medium secreted crucial for osteoblast and osteoclast differentiation.

Observation in the natural environment *in vivo* is necessary to confirm the ability to integrate and differentiate MSCs toward osteoblast-like cells. In our experience, *in vivo* examinations carried out using the rat model, we demonstrated presence of Bio-Oss® Collagen scaffold seeded with alive WJ-MSC 21 days after transplantation. In order to verify the complete integration of the bone substitute with the bone tissue of the rat, a similar observation would have to be made several months after the transplantation [37]. However, despite such a short time, after postmortem examination, it can be concluded that the scaffolds with injected cells have become more integrated with the skull bones than the empty skeleton alone, which may result from the formation of cartilage, impossible to visualize in computed tomography. Similar results were described for maxillary sinus elevation with different bone substitutes and BMSCs [38], although the low survival rate of grafted BMSCs was reported, which resulted from acute inflammation and mechanical damage [39] and indisposed to use the therapy in a clinic.

## 5. Conclusions

The reconstruction of the bone defects is a procedure that requires materials other than the mandible, long bones, or skull. By applying computed tomography, the shape of the defect can be clearly visualized. However, still the border between the graft and the patient's bone should be defaced. For this purpose, the used material must be elastic, easy to remodel, and flexible during surgery. The addition of 10% collagen in Bio-Oss® Collagen scaffold makes it formable and easy to handle. Moreover, it favours the cells to attach to the scaffold and leads to better survival and differentiation. According to our observations, the skeleton can be placed and fitted to the defect-like plasticine and then injected with cells. This will not disturb cell colonization or reduce cell survival. Thanks to the appropriate porosity, the cells will create inside local niches. On the other hand, we proved that WJ-MSC might be the optimal cell source for reconstructive therapy in clinic. They possess the same capacity for osteogenesis and secretion of factors necessary for bone formation as other MSCs, and at the same time, they are less immunogenic, which allows even allogeneic transplantation. In addition, they are more readily available and, with the appropriate isolation and culture method, their potential for survival and proliferation is very high. Presented in the manuscript graft consisted of clinically used scaffold, and the nature of WJ-MSC has a chance to be immediately used in a clinic in the reconstruction of bone defects.

## Figures and Tables

**Table 1:** Primary antibodies used for immunocytochemistry.

Primary antibody	Source	Isotype	Dilution	Company
CD73	Mouse monoclonal	IgG3	1 : 200	Dako
CD90	Mouse monoclonal	IgG1	1 : 200	Dako
Ki67	Mouse monoclonal	IgG1	1 : 400	Novocastra
Osteocalcein	Mouse monoclonal	IgG3	1 : 200	Thermo Fisher Scientific
Osteopontin	Goat polyclonal	IgG	1 : 100	Thermo Fisher Scientific

**Table 2:** 

Gene symbol	Gene name	NCBI reference gene	Primer sequence	Product length	Product efficiency *E* (%)
*GAPDH*	Glyceraldehyde-3-phosphate dehydrogenase	NM_001289745.2	F: GGTGTGAACCATGAGAAGTATGA	123	1.91
			R: GAGTCCTTCCACGATACCAAAG		
*B2M*	Beta-2-microglobulin	XM_005254549.3	F: CCAGCGTACTCCAAAGATTCA	94	1.85
			R: TGGATGAAACCCAGACACATAG		
*RPL13A*	Ribosomal protein L13a	NM_001270491.1	F: CGAGAAGAACGTGGAGAAGAAA	105	1.92
			R: GGCAACGCATGAGGAATTAAC		
*HPRT1*	Hypoxanthine phosphoribosyltransferase 1	NM_000194.2	F: CGAGATGTGATGAAGGAGATGG	98	2.22
			R: TTGATGTAATCCAGCAGGTCAG		
*TBP*	TATA-box binding protein	NM_003194.4	F: TCTTGGCGTGTGAAGATAACC	100	1.87
			R: GCTGGAACTCGTCTCACTATTC		
*PPIA*	Peptidylprolyl isomerase A	XM_024446809.1	F: GGTCCCAAAGACAGCAGAAA	115	1.87
			R: GTCACCACCCTGACACATAAA		
*BGLAP*	Bone gamma-carboxyglutamate protein	NM_199173.5	F: AAATAGCCCTGGCAGATTCC	105	1.96
			R: CAGCCTCCAGCACTGTTTAT		
*ALPL*	Alkaline phosphatase	XM_006710546.3	F: GGAGTATGAGAGTGACGAGAAAG	103	—
			R: GAAGTGGGAGTGCTTGTATCT		
*RUNX2*	Runt-related transcription factor 2	NM_001278478.1	F: TGTCATGGCGGGTAACGAT	147	1.95
			R: AAGACGGTTATGGTCAAGGTGAA		
*COL1A1*	Collagen type I alpha 1 chain	XM_005257058.4	F: GAGGGCCAAGACGAAGACATC	140	1.92
			R: CAGATCACGTCATCGCACAAC		
*VDR*	Vitamin D receptor	NM_001017536.1	F: TCTCCTGCCTACTCACGATAA	105	—
			R: GCTACTGCCCGTGAGAATATAA		
*SNAI1*	Snail family transcriptional repressor 1	NM_005985.3	F: CCACGAGGTGTGACTAACTATG	126	—
			R: ACCAAACAGGAGGCTGAAATA		

## Data Availability

The data from presented study are available from the corresponding author upon request.
